# Research Progress of Bifunctional Oxygen Reactive Electrocatalysts for Zinc–Air Batteries

**DOI:** 10.3390/nano12213834

**Published:** 2022-10-30

**Authors:** Haiyang Chang, Shanshan Cong, Lei Wang, Cheng Wang

**Affiliations:** 1 Key Laboratory of Functional Inorganic Material Chemistry, Ministry of Education of the People’s Republic of China, Heilongjiang University, Harbin 150080, China; 2 Jieyang Branch of Chemistry and Chemical Engineering Guangdong Laboratory, Jieyang 515200, China

**Keywords:** transition metal catalyst, oxygen evolution reaction, oxygen reduction reaction, zinc–air battery, catalytic mechanism

## Abstract

Zinc–air batteries (ZABs) have several advantages, including high energy density, cheap price and stable performances with good application prospects in the field of power batteries. The charging and discharging reactions for the air cathode of ZABs are the oxygen reduction reaction (ORR) and oxygen evolution reaction (OER), respectively, which play an important role in the whole performance of ZAB. Due to the cost and limited reserves of highly active precious metal catalysts, it is crucial to design alternative efficient and stable dual-functional non-precious metal catalysts. In the present review, we present a systematic summary of the recent progress in the use of transition metal-based electrocatalysts as alternatives to precious metals for the positive poles of ZAB air. Combined with state-of-the-art in situ characterization technologies, a deep understanding of the catalytic mechanism of OER/ORR provided unique insights into the precise design of excellent synthetic non-precious metal catalysts from the perspective of atomic structure. This review further shows that the hybrid electric battery is a new strategy to improve the efficiency of the hybrid electric battery, which could be available to alleviate the problem of resource shortage. Finally, the challenges and research trends for the future development of ZABs were clearly proposed.

## 1. Introduction

With the increasingly serious environmental pollution and shortage of nonrenewable energy, it is inevitable to develop and utilize renewable energies, such as wind and solar energies, in response to the calls for energy conservation and emission reduction policies. Electrochemical technology is an effective means of converting renewable energy sources from chemical energy into electricity, which is regarded as one of the most promising energy technologies [1,2]. Alternatively, rechargeable zinc–air batteries (ZABs) attract lots of attention due to the high energy density (up to 1086 Wh kg^–^^1^), abundant zinc reserves, low cost, and high safety [3,4,5]. ZABs exhibit a potential application in the power supply owing to the advantages of low cost and environmental friendliness. This is crucial to developing green energy and alleviating resource shortages [6,7,8].

Hence, as one of the most efficient energy devices, ZABs have the advantages of safety, environmental protection and high energy densities, with the potential to find applications in many fields [9,10]. The setbacks associated with lithium–air batteries, such as poor cycle stability and poor wet environment stability, can be effectively solved with ZABs, which means that ZABs have the potential to become the next generation of energy storage equipment [11]. The working principle of the rechargeable zinc–air battery is a new type of chemical power supply composed of a metal anode and air cathode, in which the 4e process of oxygen reduction (ORR) and oxygen evolution (OER) reactions are, respectively, the discharging and charging reactions of the ZAB air cathode Again, OER is a semi-reaction of decomposed water, while ORR is a semi-reaction of fuel battery. The high reversibility of the ORR and OER reactions is the key to determining the performance of the ZAB battery. With the large potential difference between the ORR and OER reactions, together with their complex mechanisms, highly efficient bifunctional ORR and OER catalysts are needed to solve their slow kinetic problem [12,13,14].

**Figure 1:**
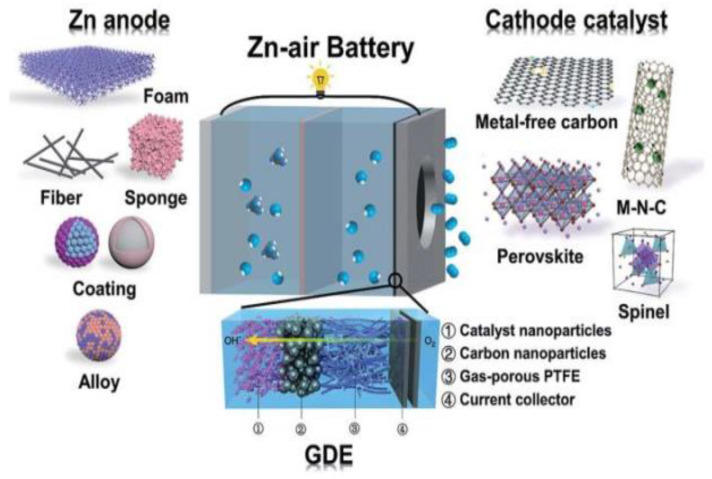
Schematic configuration of Zn–air batteries. Reprinted with permission from Ref. [25] Copyright 2019, Royal Society of Chemistry.

The development of high-performance bifunctional ORR/OER catalysts is a prerequisite for improving the efficiency and stability of ZABs [15,16,17]. At present, Pt-based catalysts are considered as catalysts with excellent ORR properties, while Ru and Ir-based precious metal catalysts have ideal OER catalytic activity [18,19,20,21,22]. However, the commercial applications of precious metal catalysts are limited by their high cost and poor stability. Therefore, the development of low-cost and efficient transition metal-based catalysts is critical to energy research. At present, many studies on the design and synthesis of non-precious metal catalysts and their application in ZABs have been reported; and some important results have been obtained. However, some important issues are still challenging, such as difficulty in explaining the internal mechanism, being unable to build a theoretical model to clarify the relationship with catalytic activity, etc. Consequently, the present review summarizes the progress on transition metal (Fe, Co and Ni)-based catalysts [23,24]. Firstly, the ORR/OER reaction (Figure 1) [25]. mechanisms were discussed, including catalyst synthesis methods, OER/ORR performances and the applications of ZABs. The catalytic mechanism was discussed using a combined theoretical calculation and in situ technology, which permitted the proposal for the design principle of a bifunctional ORR/OER catalyst. Finally, the application of zinc–air batteries was presented, together with effective insights into future developments.

## 2. ORR/OER Reaction Mechanism

### 2.1. ORR Reaction Mechanism

The positive electrodes of rechargeable zinc–air batteries are composed of two reversible reactions, namely the discharged ORR and the charged OER. Generally, ORR represents the cathodic reactions of fuel battery, while OER is the semi-reaction of the electrolysis of water. Both ORR and OER are multielectron reactions containing different oxygen intermediates (*O, *OH, *OOH, etc.). The ORR that occurs at the air cathode during the discharge process requires oxygen absorption from the surrounding air. ORR is mainly divided into two reaction pathways in an alkaline medium. In the first reaction, oxygen molecules are adsorbed on the active site (*), and they generate OH^−^ through the four-electron (4e^−^) reaction pathway, which is considered an effective method to improve the performances of ORR. The reaction equations are as follows:

(1) * + O_2_ (g) + H_2_O(l) + e^−^ → *OOH + OH^−^(aq);
(2) *OOH + e^−^ → *O + OH^−^(aq);
(3) *O + H_2_O(l) + e^−^ → *OH + OH^−^(aq);
(4) *OH + e^−^→ * + OH^−^(aq).

The second reaction is the 2e^−^ pathway, where the oxygen molecules absorb water to produce the intermediate hydrogen peroxide. But the 2e^−^ pathway is poor compared to the catalytic activity of the ORR obtained from the 4e^−^ reaction pathway. The reaction equation is as follows:

(5) O_2_ + H_2_O(l) + 2e^−^ → HO_2_^-^ + OH^−^(aq)
(6) HO_2_^−^ + H_2_O(l) + 2e^−^ →3OH^−^(aq)

Compared with the traditional synthetic H_2_O_2_ method, this pathway presents a more environmentally friendly and safe advantage [26], which can effectively control the generation of H_2_O_2_, thus providing a new direction for free radical pollution control [27].

**Figure 2:**
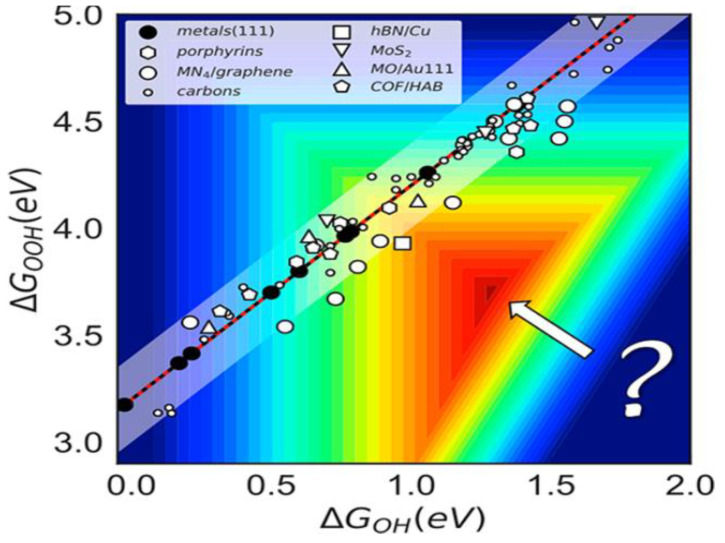
A ORR volcano plot showing the “standard” scalingrelationship (ΔGOOH = ΔGOH + 3.2) for *OOH and *OH for metals. Reprinted with permission from Ref. [29]. Copyright 2018, American Chemical Society.

The reactivity depends on the adsorption energy generated when the ORR intermediate is located in the active site. Therefore, clarifying the adsorption energy of the oxygen intermediate at each reaction step is crucial to preparing the bifunctional OER/ORR catalysts. In 1969, Balantin et al. obtained a volcanic map for the properties of non-precious metal ORR catalysts [28], and the conversion frequency of the Fe/N/C catalyst was proven to be close to that of Pt (Figure 2) [29]. Of the studied properties, the ORR limit potential value for Pt (111) was reported to be 0.8 V, while the theoretical overpotential was 0.43 V. This is considered to be the origin of the OER and ORR overpotentials. Combining *OH and *OOH with different active sites to explore the catalysts can break the scaling between them. The experiments prove that the adsorption and conversion of O_2_ to *OOH is the rapid step burst of ORR. Therefore, by reasonably regulating the free energy of the oxygen intermediates in each step, the problem of reducing the reaction rates due to the very large potential gap is avoided.

### 2.2. OER Reaction Mechanism

OER is the charging reaction for the positive poles of ZABs, and it exhibits a very complex reaction pathway and mechanism. The oxygen produced by OER originates from the metal oxidation phase but not from the metal surface. Under alkaline conditions, the reaction equation is:

(7) * + OH^−^ → *OH + e^–^;
(8) *OH + OH^−^ → *O + H_2_O + e^–^;
(9) *O + OH^−^ → *OOH + e^–^;
(10) *OOH + OH^−^ → * + O_2_ + H_2_O + e^–^;
(11) Overall reaction: 4OH^−^ → O_2_ + 2H_2_O + 4e^–^.

The desorption degree of OH^−^/H_2_O on the surface of the catalyst is crucial for the OER activity, and by improving the performance of the electrocatalytic material, the value of the zinc–air battery is fully optimized. Due to the nonrenewable and expensive nature of the commercial precious metal catalysts applied in ZAB technology, it is necessary to find ideal and cheap alternative non-precious metal catalysts to replace the precious metal-based catalysts through exploiting the mechanistic understanding of the ORR/OER mechanisms [30,31,32].

## 3. Classification of Bifunctional ORR/OER Catalysts

**Figure 3:**
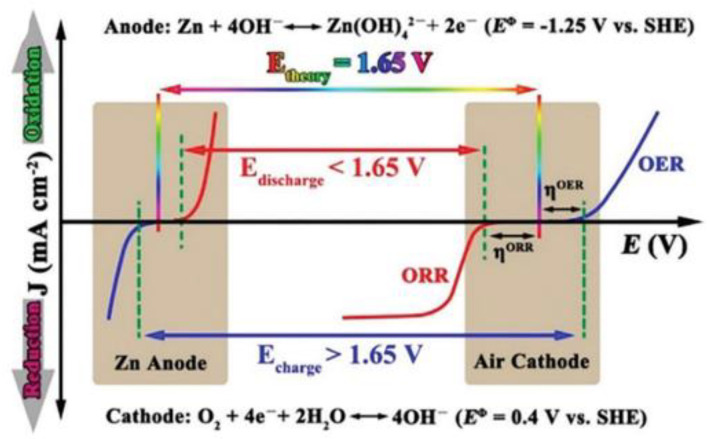
(**a**) Polarization curve of the battery. Reprinted with permission from Ref. [33] Copyright 2021, Wiley.

An ideal bifunctional catalyst requires both ORR and OER activities. At present, the oxygen electrochemical devices need to overcome the problems of slow kinetics, low efficiency and unsustainability of the ORR and OER during charging and discharging processes. It is difficult for a single element and structure catalyst to exhibit both high ORR and OER activities at the same time. For example, although the noble metal catalysts, IrO_2_ and RuO_2_, show excellent OER kinetic performances, they do not have good ORR performances. Due to the deficiency and high cost of precious metals, researchers are exploring non-precious metal alternatives to broaden the development of zinc–air batteries. The charge–discharge process of the zinc–air battery (ZAB) is shown in Figure 3 [33], and it shows that the zinc anode reacted at the standard electrode potential of −1.25 V, while the air cathode reacted at 0.4 V, and hence, the theoretical discharge potential of ZABs was 1.65 V. It is expected that, since the electron transfers for the cathode and anode reactions are too high, the overpotential between the ORR and OER should be reduced to achieve ideal states [34]. Generally, it is often difficult for catalysts to exhibit two relatively high electrochemical properties. The highest activity point was directly visible by modeling the ORR and OER dual-functional volcano [35]. The overpotential of the reaction is determined by the Gibbs free energy. From a chemical analysis viewpoint, the coordination and oxidation states of certain species in response to a specific activity also play key roles, for instance, precious metals exhibit zero valence states in the metal phase, which activates the ORR performance [36,37]. However, in a graphite environment, the binding forces of precious metals are often weak, whereas Fe is an active metal with high activity in graphite environment, and these indicate that non-precious metals are more promising and can effectively solve the current material limitation problems. Furthermore, the combination of transition metals with some graphite materials can improve ORR and OER activities. Transition metals do not exhibit fixed catalytic activities; hence, their adsorption behaviors, ambient chemical properties and oxidation states can change with the local geometry of the active site. Thus, the active sites can be designed to regulate the free energy of *OOH and *OH and then improve the catalytic properties of the two catalysts [38,39]. The theoretical model consists of four nitrogen atoms and a late transition metal, and the Gibbs free energy at each step is represented as follows:

$$\begin{array}{l} \Delta G_{1}=-(\Delta G_{\mathrm{OH}}-0.30)\\ \Delta G_{2}=(\Delta G_{\mathrm{OH}}-0.30)-\Delta G_{O}\\ \Delta G_{3}=\Delta G_{O}-(\Delta G_{\mathrm{OOH}}-0.30)\\ \Delta G_{4}=(\Delta G_{\mathrm{OOH}}-0.30)-\Delta G_{O_{2}} \end{array}$$

At present, non-precious metals often acquire a heterostructure through heteroatomic doping and defect engineering methods so as to improve the OER and ORR catalytic performances. Heteroatomic doping is one of the most widely applied methods, and a small amount of heteroatomic doping can effectively adjust the electronic structure of materials. Doping can be summarized into three methods: in situ doping, adding a variety of chemical reagents and gases, and pyrolysis of a biomass. Defect engineering is introduced to optimize the reaction kinetics and effectively reduce the band gaps of catalysts towards enhancing their conductivities. For transition metal oxides, oxygen vacancies can be introduced, and this requires sufficient edge site nanostructures for enhancing the electrocatalytic properties. Heterostructures can be better realized through the electronic structure optimizations and energy conversions than through the synergistic interactions of multicomponent interfaces. Moreover, researchers have combined heteroatom doping, defect engineering and heterostructure establishment. The coupling synergy of defect engineering and heteroatom doping helps to establish the active sites and improve the electrocatalytic activities and stabilities. For example, anchoring bimetallic atoms (NiFe) to a nitrogen-doped graphene (DG) scaffold rich in SW defects has an overpotential of up to 350 mV at a current density of 10 mA cm^−2^ and a field slope of 76 mV cm^−1^, which is comparable to the commercial Pt/C. The rich defects in nanocarbon carriers in defect engineering contribute to the regulation of the distribution of pores and improved agglomeration of metal atoms, with 85% stability at a constant voltage test of 12 h, reflecting better performances than the commercially available Pt/C [40]. Since the electrocatalytic reaction is complex, the key to improving the comprehensive performance is dependent on the improvement of the mass transfer performance. Carbon nanotubes (CNT) are found to enhance both mass transfers and the effective production of active centers; thus, efficient bifunctional catalysts can be obtained based on combined doping and heterostructures. Yang et al. adopted the mechanical force chemical pyrolysis method to synthesize a bifunctional catalyst (Co/Co_2_P@ NPCNTs) with a Moteshky heterostructure, and the unique structure of the NPCNT favored the mass transfer process, with an excellent half-wave potential of 0.88 V in the alkaline medium, as well as good stability and a power density of 189.7 mW cm^−2^ after a 200 h test. DFT calculations showed that the heterointerface of the Co/Co_2_P Motshotry structure caused an upshift of the d-band center, prompting the metal Co to move spontaneously toward Co_2_P; hence, the bifunctional ORR/OER catalytic activity was better than those of the commercial Pt/C and RuO_2_ catalysts [41]. The mechanical force pyrolysis method described herein has the combined advantages of ease of operation and greenness with relation to environmental protection, which is similar to other established methods, such as the hydrothermal method, meaning that it can be further used to prepare nano, alloy and other materials. In the actual industrial production, such Mott–Schottky electrocatalysts should be subjected to further studies in order to solve energy and environmental problems.

### 3.1. Heterojunction Structure

**Figure 4:**
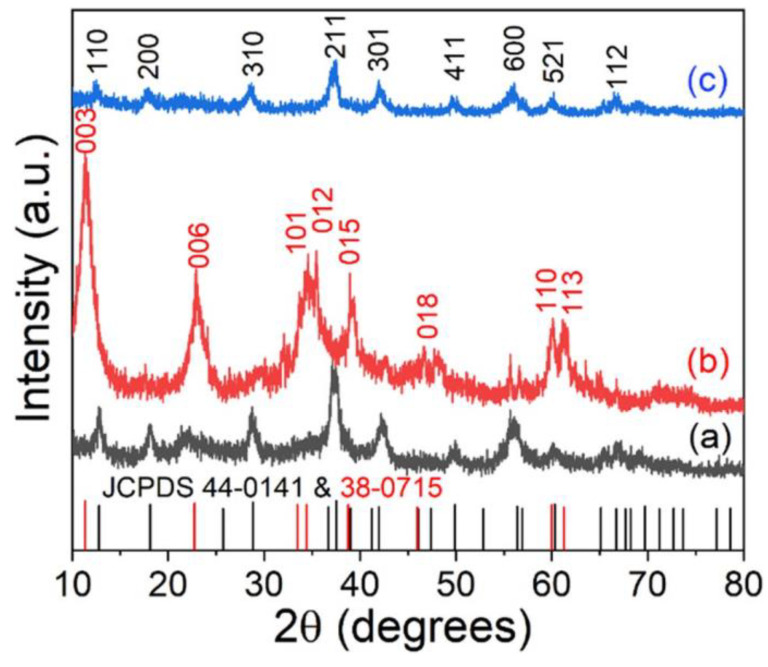
XRD spectra of a α–MnO2, b NiFe–LDHs, and c NiFe–LDHs@MnO2. Reprinted with permission from Ref. [43] Copyright 2021,Springer Nature.

Heterogeneous interfaces are designed with multiple components. Heterogeneous structures using alternate interface interactions can improve the dynamics and electron transfer speed due to the presence of different multiple phase components, and these ultimately improve the electrocatalytic performances. Therefore, the construction of heterogeneous interfaces to improve the electrocatalytic performance and preparations of bifunctional/multifunctional electrocatalysts is very crucial. Consequently, the combination of solid materials containing a transition metal (TM) with lattice-rich perovskite oxides has become a new research direction. Although TM plays a protective role on the surface of perovskite oxides, defects such as agglomeration and crystalline phase destruction still remain unresolved. In recent years, perovskite oxide (PBMNC- 0.1NS) and layered bimetallic oxide (NiFe-LDH) have been chemically connected to synthesize a two-dimensional heterojunction catalyst (PBMNC/LDH-20). Its unique structure did not only optimize the electron transfer between components but also changed the natural environment of the catalyst. The increase of the Nb (_X_) doping in PBMNC-_X_ promoted O_2_ activation, and the cycle for the charging and discharging times exceeded 100 h at a current density of 5 mA/cm^2^, which was better than the previously reported Pt/C performance [42]. In addition to perovskite, MnO_2_ with different crystal structures was also shown to react with Ni-Fe-layered double hydroxides (NiFe-LDHs), and the resulting bifunctional catalyst (NiFe-LDHs@MnO_2_) exhibited excellent ORR/OER activity (Figure 4). The unique heterostructure was effective in preventing the corrosion and oxidation of the α-MnO_2_, favoring its future application as a cathode material for zinc–air secondary batteries [43].

CoO_x_ is regarded as an efficient ORR catalyst in transition metal oxides (TMO). Although it can enhance the conductivity and stability of catalysts due to the lack of active centers, it can exploit the advantage of the synergistic effect formed with NiFe-LDH in a heterogeneous interface. Nevertheless, it is still a difficult task to combine these advantages by simple methods [44]. Recently, a new bifocal ORR/OER catalyst (LDH@N-CoO_x_@C) was reported. Its OER performance is better than that of NiFe-LDH, and this performance is similar to that of the commercial Pt/C. Its power density of 155.5 mW cm^−2^ after 450 cycles in 150 h is better than that of the commercial precious metal (Pt/C + RuO_2_) catalyst. In addition, a heterogeneous catalyst (NCS@Co/CoO_x_) was prepared using a zeolite imidazole framework (ZIF) as the template. The graded porous structure promoted mass transfer and exposed a large number of active sites, and the catalytic activity was enhanced by the synergistic effect of the Co group as the active center. As the cathode of the flexible solid ZABs, its performance exceeded that of the commercial Pt/C + RuO_2_ catalyst [45]. ZIF-67 can also be used in vulcanization reactions. Since the solvothermal and calcination reactions used in the preparation of cobalt sulfide will involve energy consumption, a one-step vulcanization strategy was developed. ZIF-67 grown on V_2_O_5_ nanorods was one-step vulcanized to obtain the V-COS/Co_9_S_8_@CNR bifunctional catalyst. The conductivity of sulfide was increased by the interactions of the two-phase interface. The coupling effect of the vanadium dopant and the coupled nanointerface accelerated the electron transport and facilitated the adsorption of H_2_O. This method of changing the metal source can be further used for the synthesis of other sulfides [46]. HQD is a heterogeneous quantum dot based on TMOs but has been rarely researched. Recently, a bifocal electrocatalyst (Co_3_O_4_-/Co(OH)_2_-HQD) was reported, and the strain effect generated by the heterogeneous interface created more active sites, improving the activity. The half-wave potential was higher than that of Co_3_O_4_ QD and Co (OH)_2_-QD following comparative experiments, and the performance was stable without degradation after 1000 cycles of testing. The battery performance of the water-based rechargeable ZABs was better than that of the precious metal Pt/C-IrO_2_-based ZABs. Since the power density in solid-state batteries can be as high as 57.4 mW cm^−2^, they possess the potential to be applied to wearable devices in the future [47].

**Figure 5:**
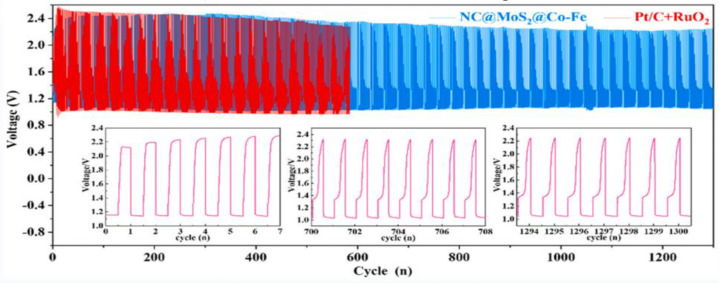
Charge (red)–discharge (blue) cycle curve at a constant current of 10 mA cm^−2^. Reprinted with permission from Ref. [51]. Copyright 2022, Elsevier.

Nickel sulfide in the transition metal sulfides (TMS) can be used as an efficient OER electrocatalytic material. It transforms high-pressure oxidation into rich oxygen vacancies (OV) in metal oxide/hydroxyl. Research proves that the conductivity of a carbon material can be improved and its energy difference shortened with the introduction of 3D transition metals to the carbon material. In order to obtain an evenly distributed TMS on the two-dimensional carbon nanotubes, Xu’s research team prepared a heterogeneous bifunctional catalyst (Ni_3_S_2_-QDs/SNC) that exhibited better catalytic performances than the currently reported nickel sulfide-based catalysts. The synergistic effect of the different components regulated the electron reconstruction interface, and the unique structure of the sulfur–nitrogen co-doped carbon support also increased the active site. The test results showed that the catalyst had a low overpotential (ORR: 0.43, OER: 0.37 eV) and a high power density (212 mW cm^−2^), which ushered in a new strategy for the development of high-performance dual-function batteries [48]. As the density of the OV increased, the number of active sites also increased. Compared with zero-dimension nanostructures, one-dimensional nanostructures can promote mass transfer and prevent serious aggregation in the reaction. This justifies the preparation of the mesoporous CoS/CoO heterojunction nanorods (CoS-/CoO-PNRs). With the unique mesoporous rod structure and the synergistic effects of the heterogeneous interface and OV, when the current density was 10 mA cm^−2^, the ORR half-wave potential reached as high as 0.84 V, and the OER overpotential was 265 mV. Hence, the battery power density and cycle life were better than those of the commercial Pt/C + RuO_2_ [49]. Electrospinning is a simple method for the preparation of one-dimensional nanofibers. Zhang used this method to produce a heterogenous electrocatalyst structure (Co_3_W_3_C/CoP/NPC) with multiple active sites and a high charge transfer capacity and power density of about 205.5 mW/cm^2^. Consequently, only 1.49 V voltage reached 10 mA/cm^2^ current density, a performance that is higher than that of the Pt/C precious metal catalyst, indicating its potential application in the heterogeneous catalytic field [50]. Molybdenum sulfide (MoS_2_) in two-dimensional layers can interact with transition metal to modify its surface electronic structure. Compared with single metals, metal alloy catalysts are more widely studied due to the valence state change that promotes conductivity changes and produces more active centers, causing improvements of the ORR/OER catalytic performances. Different metal alloy combinations in this series of action will yield different catalytic properties. For example, the electronic structure of the dual-function catalyst (NC@MoS_2_@Co-Fe) was optimized by the Co-Fe alloy, and the MoS_2_ nanosheet accelerated electron transfer and dispersion with good electrical conductivities. The carbon layer of the Co-Fe alloy effectively reduced the oxidation and passivation in electrocatalysis while improving its catalytic performances (Figure 5) [51].

Transition metal nitride (TMN) has similar properties to platinum, with a promising higher density of state and electrical conductivity. The anti-perovskite nitride (ANB_3_) shows excellent superconductivity based on the structural advantages of perovskite, but the representative Ni_3_FeN performance still requires improvements. Xu et al. synthesized a catalyst with anti-perovskite heterostructure (Ni_3_FeN/VN-NG). According to the DFT calculation results, the electron coupling generated by Ni_3_FeN and VN contributed to the charge transfer and the formation of a catalytic active center. The power density of Ni_3_FeN and VN was up to 168 mW cm^−2^, while the cycle life was over 200 h. These indicate good bifunctional catalytic activity and stability [52]. Designing the ideal electronic structure is also an effective way to improve the intrinsic activity. The surface binding energy of the electrocatalyst often directly affects the catalytic activity. However, the intrinsic relationship between the catalytic activity and the interface structure still requires more studies. The catalyst (Co_3_O_4_@Ni_2_P) prepared by combining cobalt-based material with nickel phosphide (Ni_x_P_y_) showed improved activity and stability with better catalytic activity than the commercial Pt/C + Ir/C. This laid the foundation for exploring the influence of heterogeneous structure engineering and electronic structure on the performance. In summary, the use of heterostructures with a transition metal oxide/sulfur/nitrogen synergistic effect and structural advantage can effectively improve the catalytic activity. Hence, there are futuristic needs to advance the design of the active center, the building of one-dimensional/two-dimensional/three- dimensional nanostructure and hetero-structure engineering, and to explore the nature of the interface structure and catalytic activity.

### 3.2. Defect Engineering

Defect engineering has been proven to effectively improve the catalytic activity of ORR and OER under DFT simulation. Defects can be introduced by electrochemical deposition, high-temperature pyrolysis and other methods, which can break the original electron arrangement and redistribute the charge. Point defect is a method to improve catalytic performances by local destruction, resulting in lattice distortion and changes in electronic structures. The intrinsic defects represent the intrinsic components of the crystal, while the non-intrinsic defects represent the doping and binding sites. The cathodic corrosion process can be inhibited by introducing metal elements into carbon-based nanomaterials. TM (Fe, Co, Ni, etc.) and N-co-doped carbon-based M-N-C can be used to improve the atomic utilization rate and achieve high activity and stability.TM atoms are considered to have great influence on the catalytic activity of M-N-C. At present, Fe, Co and Ni are still the main research components of M-N-C. Among the theoretical ORR activities, Fe-N-C material is the best, and Fe/Co-N-C material is the most investigated amongst the monatomic catalysts (SAC). In order to explore the effect of a penta defect on the Fe-N_4_ active center, Zeng’s research team employed a double-template method to prepare a co-doped hierarchical porous carbon (Fe/N/C-DT) with Fe-N_4_ as the active center. By comparisons with FeCl_2_-/N-/C-DT and FeSO_4_-/N-/C-DT from different iron sources, Fe/N/C-DT exhibited excellent ORR (E_1/2_ = 0.902 V vs. RHE) and OER (E_j_ = 10 = 1.66 V) performances under alkaline conditions. Its unique pentagonal structure did not only increase the surface area and active site but also enhanced the adsorption energy of the oxygen intermediates, thereby improving the catalytic performance. According to the DFT calculation results, the synergistic effect of Fe-N_4_ and pentagon carbon was beneficial to the improvement of the catalytic activity. This shows that the design using the steric effect of the iron source has application prospects in atomically dispersed active center catalysts [53]. The catalytic activity of the single atomic Fe-N_4_ site on the carbon substrate may be higher than that of the in-plane site. To verify this, the nonactive iron cluster was etched to form Fe-N_4_ in the edge position structure. The obtained bifunctional catalyst Fe/N-G-SAC had better catalytic activity and stability than the in-plane site, Pt/C and Ir/C catalysts [54]. However, the synergistic effect between the defects and Fe-N_4_ has not been clarified. Topological defects and FeN_4_ sites can be used to further improve the ORR and OER performances. The organic macromolecular ring (FePc) of Fe-N_4_ and N, P-doped defective carbon nanosheets (N, P-DC) can be synthesized by a nonpyrolytic strategy. Due to the high spin state of the iron center enhanced by the defective carbon and the charge distribution of the carbon skeleton, Fe-N_4_ can be adjusted by phosphorus doping, thereby significantly enhancing the ORR performance of the obtained catalyst FePc@ NP-DC, and this becomes better than those of the N, P-DC and the benchmark Pt/C catalysts. Additionally, the OER performance is also improved. The performance did not change significantly after 450 test cycles for 50 h, proving that the thin carbon nanosheets contributed to the electron transport and thus improved the ORR activity. Phosphorus doping can optimize the adsorption behavior and improve the catalytic activity, while the electron absorption characteristics of N and P help to capture the π electrons of FePC and enhance the stability [55]. At the same time, the prediction of the species concentration has a great impact on ORR performances. Nitrogen-modified double vacancy (ND)-deficient iron is mainly divided into the edge ND defect site (e-ND-Fe) and central ND trap site (c-ND-Fe). According to the DFT calculations, the structural advantages of e-ND-Fe can achieve four-electron ORR, and the ORR activity depends largely on the number of motifs. Under acidic conditions, Fe-N_4_/C-60 activity is comparable to that of the commercial Pt/C, which broadens the method for the rational design of defect engineering for the development of highly active transition metal atom catalysts [56]. In situ/operational Mössbauer spectroscopy is very sensitive to the structural changes of Fe-based species, which can be applied to detect the structural changes during the ORR process. The operando Mössbauer combined with XAS, and other technologies can deeply understand the catalytic mechanism from the electronic perspective [57]. Chen et al. adopted operando Mössbauer to prove that Fe^4+^ is generated in NiFe oxide transformation, and the iron oxide catalyst itself does not have Fe^4+^, so the Fe^4+^ is not the active site in the water oxidation process. Fe^4+^ under OER conditions comes from a relatively stable structure in NiOOH, although the excellent OER catalytic activity does not mainly depend on Fe^4+^, but it is still important for the role of iron in nickel iron oxide [58]. In order to deeply investigate the mechanism of Fe^4+^, Li et al. used Mossbauer spectroscopy to determine that the two sites belonged to the FeN_4_ group (high spin S1 and low/medium spin S2) in the ORR reaction and found that S1 was degraded in the reaction while S2 did not change, and only S2 still contributed to the ORR activity after 50 h of testing. Since the Fe-N-C catalyst is composed of FeN_4_ (S1 and S2), the durability of the Fe-N-C materials can improve the application in the fuel cell field by enhancing S2 or converting S1 to S2 [59].

Co atoms can be used for intrinsic defects under similar conditions, and Co atoms with similar properties can also be captured by intrinsic defects. Tang et al. prepared a triple-doped graphene-based catalyst with Co-N_X_-CCo/N/O as the active center (NGM-Co). The results of electrochemical surface activity showed that the inherent defect structure of the nanocarbon was used to generate Co-N_X_-C active sites by defect engineering. As for the ORR electrocatalyst, it exhibited a limiting current density as high as 4.74 mA cm^−^^2^ and a Tafel slope of only 58 mV dec^−^^1^. The assembled ZAB showed the maximum power density of 152 mW cm^−^^2^ at 20.0 mA cm^−^^2^, with the discharging voltage being stable at 1.19 V during the bending test [60]. The Co_9_S_8_–N-doped carbon catalyst (Co_9_S_8_ NC) with a Co-N_X_-C active center exhibited deeper defects and stronger electrocatalytic performances compared with Co_9_S_8_–C, Co_9_S_8_ and NC. The Raman spectroscopy and XPS results showed that the cooperative reaction of Co_9_S_8_ with the N-doped carbon matrix contributes to the tighter electron contact, which could present a new strategy for designing highly efficient bifunctional catalysts by combining defects with transition metal compounds and carbon materials [61]. Additionally, the 3D brush-like Co-N-C nanostructures can enhance mass transfer and electron transport, as well as the active sites; thus, it displayed excellent ORR/OER activity and cycle stability. The strategy can also be applied to Fe-based nitrogen-doped carbon analogs [62].

Single-atom catalysts (SACs) have low metal contents due to their limited synthetic conditions. Zhang et al. synthesized seven kinds of single metal SACs, two kinds of bimetallic SACs and one kind of tri-metal SAC by a formamide condensation and carbonization strategy. The bimetallic FeCoNC SACs showed much better ORR activity than the 20 wt% Pt/C under acidic and alkaline conditions, indicating its potential application for the large-scale production of SACs [63]. Thereafter, the FeCo-NC hierarchy catalyst (f-FeCo-CNT) was prepared, and its layered porous structure aided electrolyte and rapid gas passage, and this improved the electron transfer efficiency and exposed more active sites, affording a maximum power density of 195.8 mW cm^2^, and showed good long-term cycle stability at a current density of 20.0 mA/cm^2^ [64]. Despite the increasing number of studies on the combination of carbon carriers and transition metal nanostructures for defect engineering, the problems of poor chemical activity and durability resulting from issues such as the phase precipitation of surface cations in metal species remain unresolved. Wu et al. prepared the FeCo-DHO/NCNT catalyst by anchoring the Fe/Co-DHO nanoparticles on carbon nanotubes using the co-coupling effect between bimetals. The unique amorphous nanostructure inhibited the formation of crystal phases and improved the kinetics of oxygen reaction. With a current density of 60 mA cm^−2^, the voltage gap was as low as 1.085 V, and its performance was evidently better than those of most similar bifunctional catalysts. When it was assembled for all-solid ZABs, it displayed significant stability and improved the charge–discharge performances [65]. Based on previous studies, Qiao et al. combined superlarge nitrogen-doped graphene tubes (N-GT) with FeCoNi alloy to produce a highly active and durable N-GT catalyst (FeCoNi) whose OER activity exceeded that of the Ir catalyst, and its ORR activity was close to that of the Pt catalyst. Improving the difficulty of carbon catalysts to adapt to a high oxidation OER environment and using environmentally friendly water as the solvent without a template proved that ORR/OER activities mainly depend on the transition metal precursors. This provides a new direction for the application of graphene for the preparation of practical bifunctional catalysts [66].

In addition, plasma etching was also used to increase an active site and catalytic activity. A reasonable adjustment of the ratio of Co^2+^/Co^3+^ in Co_3_O_4_ produced more OV, and the specific activity of the prepared plasma-etched Co_3_O_4_ nanosheets became 10 times that of the initial Co_3_O_4_ [67]. In conclusion, the defects produced by carbon-based materials are of great significance to ORR/OER activity. The electrocatalytic activity of edge carbon atoms is higher than that of base carbon atoms. The addition of metal species Fe and Co changes the electronic structure of adjacent carbon atoms and sometimes even serves as active centers to directly participate in the reaction towards improving catalytic activities. There are still several points to be optimized for the application of defects, for example, plasma treatment as a common method of preparing carbon material defects. Aside from high production costs and limited synthesis conditions, new synthesis methods need to be developed to solve the above problems while accurately controlling the defect generation, the open numbers and dynamic changes of the reaction process require further development of in situ spectral, electrochemical and technical characterization. It is beneficial to deeply understand the influence of defects and active centers on catalytic reactions in order to provide a theoretical basis for practical applications. At present, most hybrid/metal-doped carbon-based materials cannot be applied practically, although the coupling of carbon nanomaterials to TMs is regarded as the most promising bifunctional catalysts synthesis, the chemical attachment and electrical coupling between them still need to be optimized.

### 3.3. Heteroatomic Doping

Doping is one of the effective methods of improving the intrinsic activities of catalysts by adjusting their electronic structures and local structures. At present, the commonly used method is the improvement of the activity of carbon materials by atomic doping or etching. The effective dopants in metals and nonmetals include P, S, Co, Fe, etc. Heteroatom doping is one of the most effective strategies of adjusting atomic and electronic structures. The essence of electronic structure regulation is the regulation of the active site, making the adsorption of oxygen intermediates more reasonable and reducing the barrier of the oxygen electrode reaction. This effectively enhances the intrinsic activity of the catalysts. For example, Tang et al. used the solvent heat method to produce a 3D porous co-doped vanadium nitride (VN) nanosheet assembly, and the experimental results showed that the unique 3D porous structure provided the active site and improved the specific surface area. By doping the V-rich d-electrons, Co effectively improved the intrinsic activity of VN, thus significantly improving the ORR performance. In 0.1 M KOH solution, the overpotential in the ORR was almost equivalent to that of the commercial Pt/C overpotential. After a timing current test of over 25,000 s, the current drop was very small (12%), suggesting an excellent durability. Therefore, regulating the active sites can effectively control electronic structures [68].

**Figure 6:**
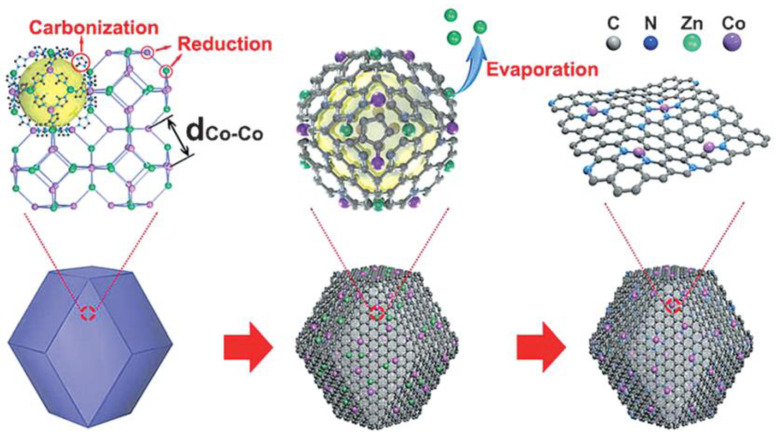
Top and bottom formations. Reprinted with permission from Ref. [75]. Copyright 2016, Wiley.

Different heteroatoms have different dimensions and electronegativity, so electronic structures can be adjusted by atomic doping. The doping of single or multivariate heteroatoms such as N and P helps to improve the electronic structures of adjacent carbon atoms. Among them, N doping changes the material structure and increases the number of active sites. The electrochemical properties obtained with higher amounts of the P dopant (the dosage) are comparable to the commercially available Pt/C and Ir/C catalysts. Recently, the electrical properties of graphene have been shown to be altered by doping, and the very large specific surface area of graphene itself was shown to improve the catalytic activity when doped with N [69]. At the same time, carbon materials with rich defective structures have also been further developed in the field of oxygen electrocatalysis. The experimental and theoretical results show that single-atom-doped OER electrocatalysts present better electrocatalytic properties compared with their undoped counterparts [70]. Huang et al. doped Mn^4+^ into a CoOOH nanosheet, and the Mn^4+^ ions increased the holes by replacing the Co position, thereby effectively modifying the electron density at the Co position, reducing the adsorption barrier of water molecules in OER and enhancing the electrocatalytic transport capacity. The Tafel slope at a starting overpotential of 195 mV was 38 mV dec^−1^, which significantly outperforms the pure CoOOH nanoplate and the IrO_2_ electrocatalyst [71]. Zhou et al. prepared Fe-doped Ni (OH)_2_ and Co (OH)_2_ nanosheets. Comparisons between them showed that Fe^2+^ doping afforded a higher electrocatalytic active surface area (ECSA), which significantly improved the catalytic activity of NiFe LDH. With an overpotential of 10 mA cm^−^^2^, current density of 245 mV and Tafel slope of 61 mV dec^−^^1^, the catalytic performance of OER was much higher than that of the original Co(OH)_2_ nanosheets. The experimental results show that the cation exchange method is effective in the design of nanostructures with enhanced electrocatalytic active sites [72]. Li et al. prepared a new Fe-doped Mn_3_O_4_ hollow egg yolk shell nanobox (Fe-Mn_3_O_4_ HYSNBs). According to the DFT calculation results, the introduction of Fe effectively adjusted the electronic structure, as well as significantly increased the number of available catalytic active sites, thus improving the electrochemical properties [73]. Lu et al. prepared a 3D nitrogen-doped porous graphene hydrogel (NHGH/NiCo_2_O_4_) using a hydrothermal method, and the doped nanocomposites showed better electrical conductivities and electrocatalytic properties [74]. Yin et al. loaded more than 4% of nitrogen-doped porous carbon on a bimetal Zn/Co metal–organic framework (Figure 6), and the half-wave potential of the Co-N_x_ sites (0.811 V) was better than that of the commercial Pt/C and most non-precious metal catalysts, indicating an excellent ORR performance [75].

Composites involving transition metals co-doped with nonmetal heteroatoms on carbon skeletons show good bifunctional activities. For example, the best ORR reaction performance was obtained by N doping. However, P has the optimal catalytic performance in OER with its low electronegativity. Co-doping of N and S can improve the electroneutrality of carbon material itself, provoke charge redistribution and improve the electrocatalytic properties of carbon materials. B and N doped with C synergistically increased the number of active sites [76,77,78]. This heteroatom doping structure helped to expose the active site and enhance the mass transfer properties of [79,80,81]. Zhao et al. prepared a porous N and S co-doped carbon (NSC) matrix with high ORR activities. The single-atomic catalysts (Fe-SAS/NSC, Co-SAS/NSC and Ni-SAS/NSC) synthesized using different metal atoms with N and S complexes had different structures, revealing Fe-SAS/NSC bonds with S and N atoms. The electronic structures of the catalytic centers of single-atom catalysts are of great significance for the rate-limiting step and the catalytic activities of the reactions, and this effectively promotes the development of ORR dynamics. According to the experimental results, the above three single-atom catalysts respectively show good ORR performances. Double doping, such as Fe-SAs/NSC, has the highest electrocatalytic performance coupled with structural advantages and not only outperformed the commercial Pt/C but also exhibited high stability after 5000 test cycles [82]. Zhang et al. prepared a new type of nonenzymatic H_2_O_2_ sensor-modified Fe_2_P with N and P co-doped carbon (Fe_2_P/NP-C). The (Fe_2_P/NP-C) material was obtained via the hydrothermal method. The Fe_2_P/NP-C material had an excellent electrocatalytic activity and can be applied in the electrochemical reduction of H_2_O_2_. This promotes the method/approach of heteroatomic-doped carbon atoms. The results show that the rational designs and optimizations of the electronic structures are key to adjusting the performances of the catalysts. In recent years, three single-atom co-coped methods have also been widely studied, and the effective electronic regulation of the active center was achieved [83]. Chen et al. prepared a hollow carbon polytope (Fe-SAs/NPS-HC) through the interactions between N, S and P to realize the electronic regulation of the active center. Fe-SAs/N-C displayed the strongest binding energy of all the intermediates, while Fe-SAs/NPS-C had the weakest binding energy of all the intermediates. According to the DFT calculations, the Tafel slope at the highest kinetic current density of 71.9 mV cm^−2^ was only 36 mV dec^−1^. Under acidic conditions, the ORR catalytic performance of FeSAs/NPS-HC was close to that of Pt/C. The unique hollow structure accelerated the ORR dynamics and was very promising in the energy storage field, which also proved that regulating the structural functions and controlling the active sites can improve catalytic activities [84].

## 4. Synthetic Method

The structures of catalysts fabricated via different synthetic methods are different. Changing the specific surface area and the surface interface properties of catalysts can affect their catalytic properties; hence, the structure of the materials can be reasonably designed and regulated to obtain the desired catalytic properties. The synthesis methods of the catalysts are mainly divided into thermal decomposition, electrodeposition, and electrospinning [85,86]. During OER/ORR, the adsorption energy of the oxygen intermediates depends on the surface geometry. In heterogeneous catalysis, the atomic arrangement of the exposed surface and the geometrical position at the interface determine the catalytic performance [87,88]. The strong interaction and unsaturated coordination site at the interface of two-dimensional materials will change the electronic structure to varying degrees, which will help to improve the electrocatalytic activity of the catalysts [89]. Therefore, the synthesis of effective structures such as the hierarchical interface and carbon-coated structures can enhance the activity and durability of catalysts [90].

### 4.1. Thermal Decomposition

Thermal decomposition is a synthesis method for obtaining a target catalyst via the thermolysis of single-molecule precursors under certain conditions. Metal–organic framework (MOF) obtained through metal and organic ligand coordination is a network of porous materials that can be used to build complex nanomaterials with improved electrical conductivities and stabilities. The combination of MOFs with carbon nanotubes is an effective method for the preparation of bifunctional ORR/OER catalysts [91,92,93]. Wang et al. prepared a three-dimensional carbon nanotube assembly (3D-CNTA) composed of a new MOF induction strategy with a rich hierarchical porous structure via pyrolysis. The hierarchical porous structure of the 3D-CNTA improved the utilization of the active site [94], while the CNTs in turn exhibited a high electrocatalytic activity. Furthermore, bifunctional electrocatalysts for the pyrolysis of ZIF-67 films on a 3D macroporous polymer substrate provided both ORR and OER activities and stabilities [95].

**Figure 7:**
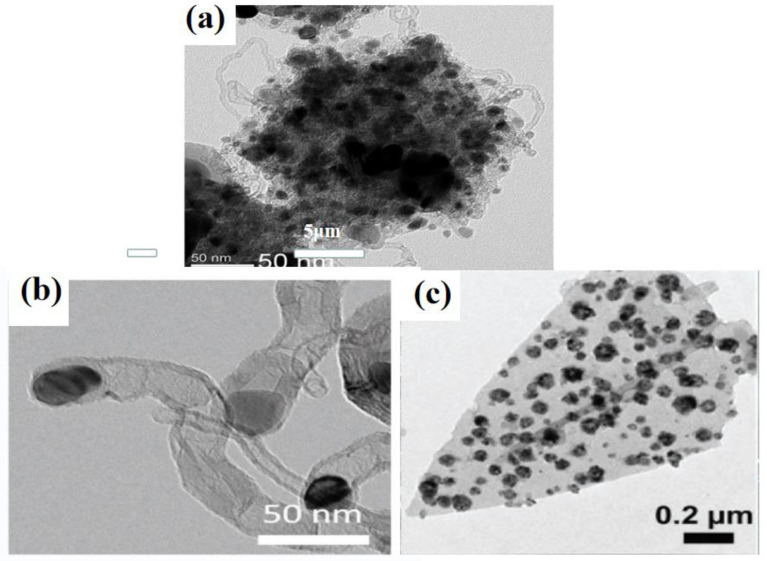
(**a–c**) TEM and high-resolution TEM images Reprinted with permission from Refs. [96,97,98]. Copyright 2016, Wiley. Copyright 2018, Wiley. Copyright 2018, Wiley.

The zeolite imidazole skeleton (ZIF) has become a hot research topic material with high porosity and high thermal stability. The particles of cobalt metal–organic skeleton (ZIF-67) maintain their original form in hollow structures and have layered shells connected with crystalline NCNTs. The ZIF-67 particles in pyrolysis synthesis not only provide the required C and N sources for catalyzing the growth processes of NCNTs, but they also serve as templates to form empty skeletons. A. Aijaz et al. obtained an oxygen electrode electrocatalyst by the pyrolysis of ZIF-67 in H_2_O_2_, and Co@Co_3_O_4_ nanoparticles with nuclear shell structures were embedded in the N-doped C polyhedra grafted by carbon nanotubes (Figure 7a). The reversible overvoltage was up to 0.85 V at 1.0 M KOH, making it one of the best choices for reversible oxygen electrocatalysts due to its superiority over Pt/C, IrO_2_ and RuO_2_ [96].

MOFs can be used as single precursors to constitute NCNTs. Wang et al. used a two-dimensional bimetal (Co/Zn) and zeolite skeleton (ZIF-L) to prepare Co nanoparticles for the packaging of nitrogen-doped carbon nanotubes based on a reasonable Co/Zn molar ratio. The highly dispersed cobalt nanoparticles were completely encapsulated at the tip of the N-C nanotubes, forming a highly active Co-N-C group, which became an excellent catalyst for a dual-function air cell (Figure 7b). Experimental results show that the ORR and OER of the Co-N-C nanotubes were better than those of the commercially available Pt/C, IrO_2_ catalysts and most of the already reported metal–organic skeleton-derived catalysts [97]. In addition to the MOF derivatives, there are other effective methods that can be used to prepare bifunctional OER/ORR catalysts with layered porous structures. H. Zhang et al. grew mesoporous layered sheet array (FAs) structures on a flexible carbon cloth, with combined (N-doped C) FAs, nanoscale P-doped CoSe_2_ hollow clusters and atomic-level P-doped P-CoSe_2_/N-C FAs. Empty nanoclusters existed in P-CoSe_2_, and the structure of the scale array on the carbon cloth was well-maintained. Se, Co, N, C and P were dispersed evenly in the P-CoSe_2_ hollow nanocluster, indicating that the doped N and P were evenly distributed throughout the hollow structure (Figure 7c). According to the DFT tests, P doping can reduce the free energy of the rate-limiting step in the OER, while P-CoSe_2_/N-C FAs can be used as the cathode of ZABs. Excellent OER and ORR electrocatalytic activities and good cycle stability in alkaline electrolyte were obtained, revealing its potential application in the bifunctional electrocatalytic field [98].

A heterostructure is composed of more than one component, and the conductivity of each component can be enhanced, while doping improves the electrocatalytic performance of the material by regulating the highest occupied molecular orbital and the lowest unoccupied molecular orbital levels. Therefore, it is necessary to conduct intensive studies on chemically doped heterostructures. For example, Liu et al. prepared a porous NiCo_2_O_4_/Co_5_._47_N/NF heterostructure nanochip array (NiCo_2_O_4_/Co_5.47_N) mixed with Mo via pyrolysis. Mo effectively reduced the free energy of the chemisorbent and thus accelerated the electrocatalytic process. The obtained unique electronic structure and the fully exposed active site gave rise to excellent electrocatalytic properties. The Mo, Co, Ni and N elements were evenly distributed on the 2D Mo-NiCo TMOF nanosheet array, and the morphology did not change significantly after thermal annealing, indicating that the nanoporous structure contributed to material migration and increased the electrocatalytic activity. Mo-NiCo_2_O_4_/Co_5.47_N/NF produced a current density of 50 mA cm^−2^ at low overpotentials of 310 mV and 170 mV, and when applied on water decomposition batteries, the current density reached 10 mA cm^−2^ at a low potential of 1.56 V, which is an excellent catalytic performance [99]. Yuan et al. doped B element in the Co-N-C single atom catalyst to form an electron defect site through thermolysis. The activation of the Co-N-C site enhanced the electron transfer and consequently accelerated the OER and ORR kinetic responses of 4e^-^ processing. The DFT results showed that the coupling of the Co-Nx active site and the B atom caused the adsorption of unilateral O_2_ molecules, thereby accelerating the ORR dynamics [100]. A series of studies have also been carried out on bimetal monoatomic catalysts. For example, a dual Co-Ni site catalyst (CoNi-SAs/NC) was obtained by the pyrolysis and coating of dopamine CoNi-PBA [101]. The study explored the role of mono-atom and diatom metal structures in promoting ORR/OER activities and proposed that the trend of ORR capacity was NC < Ni-N < Co-Ni-N. The synergistic effect of the uniformly dispersed single atoms with adjacent Co-Ni bimetallic centers optimized the adsorption and reduced the reaction hindrances. However, the metal particles in single-atom catalysts are prone to agglomeration due to the need to shrink to the atomic level, prompting excessive surface energy. Therefore, the selection of single atoms and carriers is uneasy, thereby affecting the realization of the goal of preparing single-atom catalysts on any carrier and metal.

### 4.2. Electrodeposition

The electrodeposition method can be used to prepare different electrode materials, with the advantage of controlling both the composition and morphology. The structure of the electrode material in a solid–liquid two-phase interface determines the catalytic properties, so the study of the surface properties of materials is crucial. He et al. synthesized a CoP (2D) nanochip array (NSA) and amorphous FeNiLDH nanosheets on a flexible carbon cloth (FeNiLDH/CoP/CC). Due to the positive center of the charge on the surface of the Fe-Ni-LDH, electron transfer occurred when the n-type Fe-Ni-LDH was coupled with the p-type CoP. According to the DFT calculations, the adsorption energy of the Fe-Ni-LDH surface alone was higher than the performance of the OH-fold NiFe_2_O_4_/FeNi_2_S_4_ at the CoP surface coupled with Fe-Ni-LDH [102]. The combination of electrodeposition and hydrothermal methods can be used to design highly efficient 3D nanochip arrays, for example, Liu et al. prepared three-dimensional nanosheets (CoFe@ NC/CC) on a carbon cloth with nitrogen-doped carbon-bonded CoFe alloy nanoparticles. The DFT calculations showed that the introduction of the N-doped C structure effectively reduced the O_2_ adsorption energy of the CoFe alloy nanoparticles, and the 3D structure provided more active sites during the oxygen reaction, thereby improving the ORR/OER bifunctional catalytic activity [103].

In addition to transition metals, highly active non-noble metal ORR catalysts were directly synthesized by electrodeposition and the pyrolysis of carbon powder in the presence of dicyandiamide. The obtained CO_2_-derived catalyst not only has higher activity but also has higher stability [104]. Remmel et al. collected from atmospheric and exhaust gases and analyzed the effects of the structure and synthesis conditions using an accurate physical analysis such as XRD. Based on the physical and catalytic properties of synthetic carbon materials, a reasonable structure and activity relationship has been established, and the bifunctional catalyst method can be used as a new sustainable catalyst to mitigate high carbon dioxide emissions in the future. We can further study the bifunctional OER/ORR catalyst without a carbon dioxide footprint to realize the green transition from a carbon economy to a hydrogen economy [105]. Lacarbonara et al. used CO_2_-captured graphite as the anode and transition metal oxide as the cathode. The graphite phase showed good catalytic performance after environmentally sustainable chemical treatment, which could bring up a new direction for carbon capture and storage, expecting to prepare materials that can reduce the CO_2_ concentration and alleviate the greenhouse effect in the future [106]. In summary, the electrodeposition method is an important strategy for the preparation of the cathode catalysts for ZABs with several advantages, including: low cost, high efficiency and precise regulation of the chemical composition and structure.

### 4.3. Electrostatic Spinning

**Figure 8:**
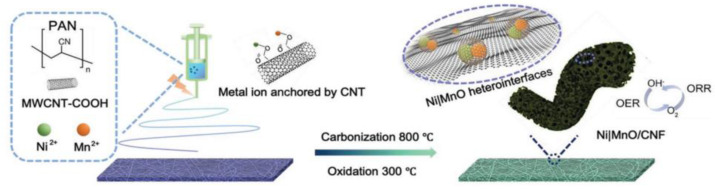
Illustration of the fabrication process. Reprinted with permission from Ref. [109]. Copyright 2020, Wiley.

Electrospinning technology uses structural regulation and interface engineering to regulate the fiber arrangement, increase the contact area between the catalyst and electrolyte and improve the interactions between them, thus promoting the ORR and OER reactions. In this regard, Pei et al. prepared (a) a 1-dimensional fiber electrocatalyst with a bamboo structure, which showed the lowest reversible overpotential of 0.7 V, and (b) synthesized a unique non-noble nitrogen–carbon (M-N-C) catalyst using coaxial electrospinning technology. The polarity of the end group played a crucial role in the antifreeze properties of the hydrogel, and the ZABs exhibited excellent mechanical flexibility and stability when the operating temperature dropped from 25 °C to −20 °C [107]. The combination of the electrospinning technology with other auxiliary methods can effectively adjust the activity barrier of the OER/ORR bifunctional catalyst. This presents great development and economic potential. Pan et al. prepared CuCo_2_S_4_ nanosheets @ N-doped C nanofiber (CuCo_2_S_4_NSs@N-CNFs) films using electrospinning and room temperature in situ sulfation. An excellent bifunctional catalytic performance (E_j_ = 10 (OER) − E_1/2_(ORR) = 0.751 V) was recorded with the capacity maintained at one thousand times the bending experiments from 0° to 180° and showing broad application prospects [108]. Ji et al. prepared Ni|MnO/CNF catalysts by electrospinning calcination. Employing the strong interactions between Ni/Mn alloys, the synergy between the Ni|MnO heterojunction interface and the one-dimensional porous CNF carrier exhibited excellent ORR and OER catalytic activities. The Ni|MnO/CNF catalyst exhibited the lowest reversible potential (0.763 V) at 0.1 M KOH. This makes it one of the best active bifunctional oxygen catalysts, and it also displayed a cycle life beyond that of the precious metal ZABs (Figure 8) [109]. However, the current heat loss problem associated with electrospinning technology means that there is still a need to further explore organic/inorganic hybrid composite fibers with the requisite properties suitable to improve the mechanical stability energy of fiber products.

### 4.4. Other Methods

The oxygen catalytic reaction of the ZABs is regarded as a heterologous catalytic reaction at the three-phase reaction interface, which requires enough active sites for contact with reactants to expand the reaction areas, as well as a stable three-phase interface of a gas, liquid and solid to achieve a sufficient catalyst reaction [110]. Reasonable regulation of the interface electronic structure reduces the reaction energy barrier and enhances the reactivity [111]. The hydrothermal method is a wet chemical method in closed containers, which can be used to prepare catalysts for heterogeneous interfaces. Compared with other methods, it is characterized by mild reaction conditions, simple operations, high crystallinity and high purity. Lu et al. constructed MnO/Co heterointerface in porous graphite carbon (MnO/Co/PGG) by hydrothermal calcination. The Co nanocrystals displayed excellent ORR and OER catalytic activities and stability with good heterointerface and high electrical conductivity. MnO/Co/PGC driven ZABs can stably charge and discharge more than 350 times more than the Pt/C||RuO_2_-driven ZABs, demonstrating that synergy in the heterogeneous interface is crucial for improving oxygen electrocatalytic activities [112]. Huang et al. grew new Ni-N-O porous interface nanoparticles (NiNO-INPs) through in situ oxidation, and the strong interaction between Ni_3_N and NiO further enhanced the performance of the electrocatalytic OER. The strong coupling nanointerface improved the efficiency of electrolysis by about six times. According to the DFT results, the introduction of a biomimetic structure into the electrocatalyst led to a full identification of the active sites and improvement of the OER catalytic performance [113]. Therefore, the ideal electron path and fully exposed active center can be achieved by optimizing the interface of the nanostructure with the conductive carrier. 

The properties of the material at the interface were significantly influenced by the strong interactions between the matrix and the enhanced body in the high-energy heterojunction interface structure. Although the study of the heterostructures have made considerable progress in recent times, it is still confronted with some challenges, such as (a) reduced reversibility of deposited ORR products on the electrode surface, (b) enhanced corrosion of the glass carbon electrode due to the high electric potential and (c) the large gaps between theoretical activities and actual performances. Since metal assemblies mostly grow singly, the heterojunction method is usually used to transform metal parts into metal compounds. Through the postprocessing of bimetal (oxygen) hydroxide, a tightly bound interface is formed to reduce the resistance. For example, Yin et al. prepared high-performance zinc–air battery catalysts by in situ electrochemistry on oxygen-dominated porous nanowires at a cobalt–nickel sulfide interface (NiS_2_/CoS_2_-O NWs). The NiS_2_/CoS_2_-O NW catalyst dominated by an oxygen vacancy in the zinc–air battery had an excellent performance. The positive and negative electrodes of the self-driven water separation device were powered by the portable ZAB, which is in line with the concept of efficient chemical energy conversion to electrical energy [114]. Li synthesized NiO/CoN a porous interface nanoarray (NiO/CoN PINW) via in situ nitride. Due to the strong coupling of oxygen vacancy and the NiO and CoN nanointerface, the stability and electrocatalytic properties of the ORR and OER were improved, and the low-cost and high-performance transition metal-based bifunctional electrocatalyst showed possibilities of practical applications [115]. The advantages of the two metals can also be combined by electrostatic stacking to prepare a heterointerface structure catalyst [116]. For example, through an electrostatic stacking method, the heterogeneous structure of the Ni-Fe LDH nanosheet @ the defect graphene composite (NiFe LDH-NS@DG10) was coupled where the 2D NiFe LDH layer was rich in highly active defects, and the high conductivity graphene supplied the anchor site. Heterostructural hybrid materials constructed from two-dimensional materials are optimized by direct interface contact [117]. An et al. synthesized NiFe_2_O_4_/FeNiS_4_ heterostructured nanosheets (HNSs), which have highly efficient OER and ORR catalytic properties. The uniform distribution of FeNi_2_S_4_ nanodomains on the NiFe_2_O_4_ NSs surface demonstrates the successful construction of the NiFe_2_O_4_/FeNi_2_S4 heterostructural interface. The DFT calculations showed improvements in the OER and the ORR performances due to the abundant oxide/sulfide interfaces formed on the NiFe_2_O_4_/FeNi_2_S_4_ NHSs surface. A power density of 44.4 mW cm^2^ was produced when the material was used as an air electrode, and the most efficient ZABs are those synthesized in neutral aqueous solution [118].

Single-atom catalysts (SACs) are atomic-scale catalysts with excellent atomic utilization rates and electrocatalytic activities with their special structures. For the first time, Qiao et al. reduced the particles to a single atomic scale for the electrocatalytic reaction by preparing a single-atom catalyst and propounding the concept of “single-atom catalysis” [119]. Ji et al. used the “impregnation carbon-carbonization acidification” method to prepare an atomic dispersed TM-SA (TM = Co, Ni) ORR/OER bifunctional catalyst whose main body was carbon nanofibers, and it was used to support N-doped porous carbon sheets (M SA@NCF/CNF) anchored by metal monoatomic sites. The single atoms supported by the N-doped carbon sheet arrays grown on the carbon nanofiber assembly (M SA@NCF/CNF) had high catalytic activity. The independent graded porous structure effectively enhanced the accessibility of the active sites with long-term durability in alkaline solutions, placing it over most of the reported advanced bifunctional oxygen electrocatalysts [120]. Recently, Qiu et al. prepared Ni and N co-doped 3D nanoporous graphene through the chemical vapor deposition (CVD) process and chemical etching. The product finds application as an excellent dual-functional catalyst in alkaline environment and can perform more than most reported bifunctional oxygen electrocatalysts. The excellent performance of the commercial Pt/C was achieved by fixing the high loading amount of single Ni atoms in the N-doped nanoporous graphene. According to the DFT results, the synergistic effects of N and Ni promoted the ORR and improved the electrocatalytic activity by improving the binding energy [121].

The structure and morphology of ZIF-67 are greatly influenced by its synthesis conditions, and its growth orientation is mainly dependent on the reaction solvent. For example, Zhong et al. creatively designed the air cathode structure of MOF-on-MOF and synthesized a flexible carbon cloth anchored by multilayer Co_3_O_4_ nanoparticles in a nitrogen-doped carbon nanoarray through high-temperature carbon oxidation (Co_3_O_4_@NCNMAs/CC). The layered structure was effective in loading multiple electrocatalytic active sites, which significantly improved the energy density and reaction dynamics of all-solid ZABs. The Co_3_O_4_ nanoparticles exhibited an angular and rough morphology in the nitrogen-doped carbon nanomicroarrays (ZIF-D-Co_3_O_4_). The Co_3_O_4_ nanoparticles were evenly embedded in the 3D and 2D carbon matrixes (ZIF-D-Co_3_O_4_ and ZIF-L-Co_3_O_4_), and the Co-MOF-derived N-CNMAs effectively prevented the aggregation of the Co_3_O_4_ nanoparticles, thus promoting electron transfer. Moreover, the resulting synergistic effect enabled OER and ORR to show both good electrocatalytic activity and stability [122]. In contrast, the H_2_O_2_ oxidation treatment method is simple and can effectively regulate the degree of oxidation and can also be used to explore the role of surface sulfur residues in the catalytic activity of metal (oxygen) hydroxides. Cho et al. prepared a sulfur-containing NiFe (oxygen) hydroxide with a high OER activity at 1.0 M KOH. The Ni_6/7_Fe_1/7_-OH-_6_/CNT catalyst showed a better durability than the Pt/C + IrO_2_ hybrid electrode after 150 cycles. According to the DFT calculations, the free energy adsorption of the OER intermediate at the Fe site can be optimized by the surface S residues [123].

In summary, (a) hydrothermal and H_2_O_2_ oxidation treatment, immersion-carbonization acidification and chemical vapor deposition processes can be used to synthesize single-atom catalysts; (b) high-temperature carbon oxidation can be used to synthesize multilayered structure catalysts; (c) electrostatic stacking and wet chemical sulfation can be used to build a heterojunction interface and (d) in situ methods such as in situ oxidation, nitration and electrochemistry are used for preparing efficient and stable bifunctional catalysts.

## 5. Research on Catalytic Mechanism

In situ technologies are emerging detection methods that have become increasingly popular in recent decades. Combined with the multiscale theoretical simulation and calculation method, we can obtain more direct and accurate information about the actual reaction process, which can be used to explain the reaction mechanisms. In heterogeneous catalytic research, in situ Raman, in situ FTIR, in situ XRD, in situ XPS, in situ XAFS and in situ XAS are commonly used. Among them, in situ Raman technology is used for group analyses and characterizations of molecular structures, thereby permitting the identification of the bonding modes of atoms in the molecules. The common in situ Raman spectrum is surface-enhanced Raman spectroscopy (SERS) and shell-isolated nanoparticle-enhanced Raman spectroscopy (SHINERS). In situ infrared technology is often used to characterize the variations of substances with high dipole moments, which can effectively track the active states of adsorbed species on the surfaces of catalysts. It can also effectively identify the compounds and group molecules involved in reactions. For the characterization of groups and molecular space configurations, the combination of in situ Raman and in situ infrared is often adopted. In situ XRD is used to detect the changes in the material and material phases during reaction processes, and it has the advantages of being nondestructive and fast, as well as possessing the ability to analyze a range of data. The identification of surface species before and after H_2_ treatment in fixation systems can be achieved using in situ XPS, including the effective monitoring of the time- and temperature-dependent changes in the catalysts. In situ XAFS mainly consists of single scattering and multiple scattering theories and can be used to gain deep understandings of the kinetic data obtained at steady and instantaneous states. Changes in electrode materials during ion extraction/insertion can be carefully tracked with in situ XAS, contributing to deeper insights of the correlations between structures and electrochemical properties. In situ FTIR is a combination of electrochemistry and infrared spectroscopy. FTIR is very sensitive to the changes of polymer structures. On this basis, in situ FTIR can also effectively monitor the electrochemical parameters and the active species on the electrode surface, which can be used to explain the electrocatalytic reaction mechanism from the molecular level. In order to deeply explore the electrocatalytic process, Lv et al. combined with in situ FTIR and experimental research expounded on the synergy and the structures of highly active species at the molecular level, benefitting from deeply exploring the internal source of the activity and the potential electrocatalytic mechanism [124]. Hence, in situ technologies are highly effective in providing characteristic information of catalytic reactions during reaction processes, and the combination of these test techniques can aid in the understanding of the whole catalytic reaction process, thus providing reasonable scientific evidence for the development of new catalysts.

SHINERS spectroscopy belongs to surface-enhanced Raman spectroscopy (SERS) class, which is a nuclear and shell structure covered by the surface deposition protective layer of plasmon nanoparticles. It can effectively block nanoparticles from contact with the measured samples and inhibit agglomeration, as well as weaken the electromagnetic enhancement effects. Yan et al. used in situ Raman spectroscopy and XRD to describe the possible adsorption phenomena on carbon materials during a battery operation, and they qualitatively studied the changes in the activity center. They proved that dopants can effectively improve ORR activity, and they explored the reaction mechanism of heteroatomic-doped carbon in ZABs [125]. Wang et al. used SHINERS to explore the ORR processes of bimetallic Pt_3_Co catalysts in acidic and alkaline environments and revealed the importance of the reactive oxygen intermediates. According to the DFT calculations, the weak *O adsorption occurring on the catalyst surface promotes ORR activity. The reaction intermediates in the solution can be directly detected, so that the possible evolution of the original platinum surface can be monitored, and the association mechanism of the Pt_3_Co nanocatalyst in the ORR reaction is proposed [126]. Burke et al. determined that, through in situ electrochemical measurements, the addition of Fe can enhance the OER activity. The inherent OER activity of FeOOH itself was higher than that of CoOOH. CoOOH can serve as the host of Fe conductivity and electrolyte penetration, when Fe and Co were strongly coupled in solids, and an effective optimization for the catalyst design was achieved [127].

**Figure 9:**
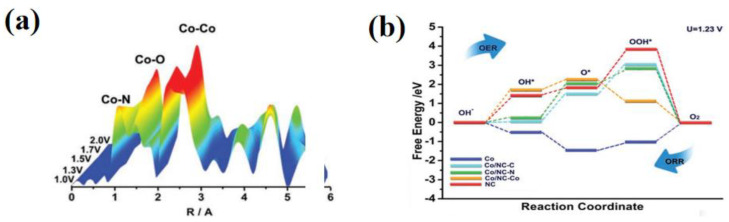
(**a**) The bond length of Co K-edge increases from low (blue) to high (red) voltage. (**b**) Different free energies obtained. Reprinted with permission from Ref. [132]. Copyright 2019, Wiley.

For the first time, Deb et al. employed the in situ XAS to monitor the changes in the electrode materials during the charging and discharging cycles. The EXAFS and XANES results revealed that the changes in the electrode materials were minimal [128]. Jia et al. provided strong evidence for the conversion behaviors of different active sites in ORR through in situ XAS. It proposed that the combination of the dynamic structure of the M-Nx-C site with its catalytic activity can be used to the design nonplatinum group metal [129]. Shi et al. designed and synthesized a single-atom catalyst (PCN-A@FeSA) with a porous carbon nanocubic structure, and it was inferred from the XAS characterization that Fe^2+^N_4_ was the main existing form of the single-atom catalyst. This structure helped to improve the affinity of oxygen, as well as the detachment of the OH* intermediate, making it ORR catalytically active [130]. Seo et al. demonstrated by in situ XAS analysis that CoO_x_ NPs had auxiliary effects on the ORR, but the size of its particles did not influence the ORR activity [131]. Yu et al. used Co-N-C nanosheets that were grown with C-felt cloth to synthesize Co nano-islands with a 3D hierarchical structure (Figure 9a). The unique structure facilitated electron/ion transport and thus enhanced the ORR/OER bifunctional electrocatalytic activities. The voltage did not change significantly after the 10 h battery cycle (Figure 9b), indicating that the catalyst was stable. In terms of the adsorption energies of OH*, O* and OOH* intermediates at the C position, the properties of Co/NC were significantly lower than those of an NC material. It was demonstrated that the C active site in the Co-N-C structure acted on the ORR, and that the Co active site favored the OER. According to the DFT calculations, the presence of the Co-N-C active site between the Co and N-doped carbon interfaces favored the formation/deposition of O* and OOH* intermediates in the rate-determining steps during ORR and OER, revealing the origin and the dominant mechanism of the two oxygen active sites in the Co/Co-N-C bifunctional catalyst [132]. 

**Figure 10:**
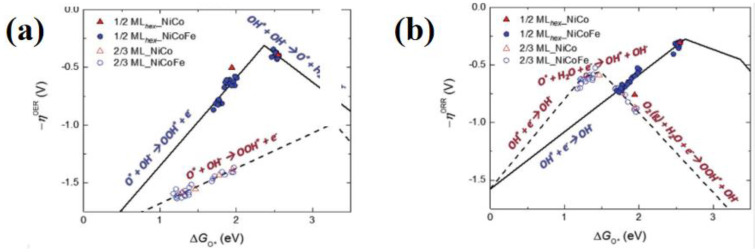
(**a**) Volcanic map of OER, and (**b)** ORR synthesis roadmap. Reprinted with permission from Ref. [135] Copyright 2018, Wiley.

Recently, iron-based catalysts generally showed high catalytic activities for the ORR, while cobalt-based catalysts showed more common applications for the OER. Therefore, the design of an advanced bifunctional catalysts can be achieved by combining the synergy of the two metals (iron and cobalt). For example, Liu et al. reported the embedding of CoFe alloy NPs in nitrogen-doped bamboo carbon nanotube structures, and FT-EXAFS spectra demonstrated that ORR processes occurred mainly on the Fe surface, while OER processes dominated the Co surface. The synergy between Fe and Co contributed to the improvement of the bifunctional catalytic activity. The reaction mechanism of the FeCo-based catalyst for OER/ORR was deeply studied, and a new method for developing high-efficiency and high-performance oxygen electrocatalysts was birthed [133]. Deng et al. used bimetallic nitrides as the main body to monitor the structure evolution of (Co, Fe)_3_N_R during the charge and discharge by in situ XAS. During the initial discharge process, the gradually maturing shell changed the shell structure of nitride into hexagon hydroxyl hydroxide. In the ripening process, the transformation of the active site increased the active area that was expanded by the hydroxyl shell, making the power density of the battery reach 234 mW cm^−2^. This proposes the concept of a dynamic electrocatalyst [134]. Cho and others prepared a carbon-coated Ni_46_Co_40_Fe_14_ nanoalloy (C@NCF-900) as an ORR/OER bifunctional electrocatalyst. The catalytic activity was higher than those of Pt/C 20 wt% and the IrO_2_ catalysts, and there were no significant changes in the voltage before and after 1000 cycles. XAS provided the reaction mechanism of the NiCoFe nanoalloy electrocatalyst (Figure 10a,b) [135].

Bates et al. tested the activity of binary and ternary mixed metal oxide (MMO) membranes with XAS, in which Co locally shrunk the geometry of Ni and Fe and reduced the spacing of the central bonds of Ni, finally proving that Fe is the active site for OER, and its mass activity is optimal among the currently known Fe-based catalysts. Compared with Ni, this catalyst exhibited excellent mass activity and had the highest Fe-based OER activity reported to date. There was no significant difference between the OER activity and the NiO phase on the MMO surface hydration Ni(OH)_2_ phase, and the excellent dispersion obtained can be attributed to the unique morphology of the catalyst membrane on the Ni carrier [136]. Friebel et al. used XAS technology to prove that Fe^3+^ is located in the octahedral position of Ni_1−x_Fe_x_OOH. According to the DFT and U calculations, it is known that Fe is the active center of Ni_1−x_Fe_x_OOH, and the addition of γ-NiOOH reduced the overpotential, thus improving the OER activity [137]. Since the nature of the active center of nickel–iron oxide is still not defined, Hu et al. obtained different active sites of Fe-free and Fe-Ni oxide by ^18^O isotope-labeling combined with in situ Raman, and they determined that lattice oxygen existed in the OER processes of Ni and NiCo-LDHs but not in NiFe and NiCoFe. The addition of Fe formed a new active site and significantly improved the catalytic performance [138]. Lee et al. further explored the origin of Fe activity in NiFe-LDH, and through the combination of oxygen isotope labeling and Raman spectroscopy, it was found that Ni released oxygen with the participation of lattice oxygen, while Fe generated oxygen without the participation of lattice oxygen, supporting the idea that Fe depends on the activity of NiFe-LDH [139]. In summary, a variety of in situ characterization techniques were useful to gain a deeper understanding of the internal and chemical mechanisms of electrode materials and interfaces during the charging and discharging processes, and they provided the theoretical basis for the data analysis of the next generation of ZABs.

## 6. Application of Zinc–Air Battery

**Figure 11:**
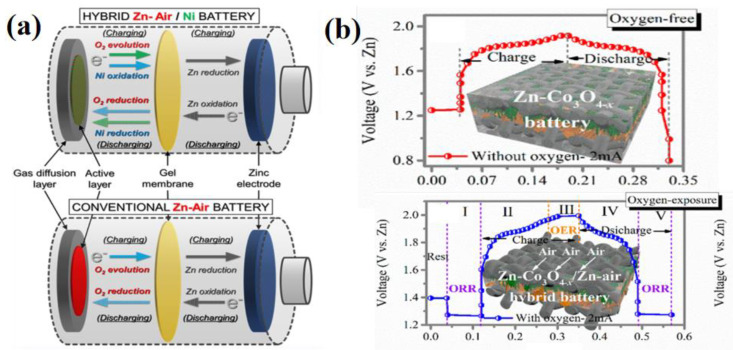
(**a**) Concept of the zinc/nickel hybrid rechargeable battery.Reprinted with permission from Ref. [140], Copyright 16, The American Chemical Society. (**b**) oxygen-free and oxygen-exposure conditions. Reprinted with permission from Ref. [141] Copyright 18, The American Chemical Society.

Although rechargeable zinc–air batteries have been constantly making breakthroughs, their existing problems, such as low energy efficiencies and short cycle lives, have not been fundamentally solved, so it is necessary to develop new hybrid ZABs with high efficiency and good stability for practical applications. The Zn–Ni/Zn–air hybrid battery uses a NiO/Ni(OH)_2_ mesoporous ball as the active material. The hybrid battery works based on two different battery reactions and shows a significantly better power density and energy density (980 W h Kg^−1^) than the other hybrid battery, and the charging speed is ten times the discharge speed (Figure 11a). The batteries are a single electrochemical battery and are of different types, and they simultaneously meet the high current and energy density demands. It is a low-cost hybrid energy storage device. This strongly proves the concept of the new hybrid charging battery, which is expected to be mass produced and used in hybrid electric vehicles in the future [140]. Ma and others built a Zn−Co_3_O_4−x_ and Zn–air hybrid battery system with cobalt oxide etched by Ar plasma as the cathode. The Co_3_O_4−x_ had various electrochemical reactions and strong environmental adaptability, with excellent OER and ORR performances (Figure 11b). The hybrid battery power reached 3200 W kg^−1^, qualifying it as a flexible electrode for underwater application [141].

Furthermore, the hybrid battery material, MnCo_2_O_4_/NGr, prepared on nitrogen--doped reduced graphene oxide, had an ORR activity comparable to Pt/C, with an additional excellent OER activity. The cycle efficiency of MnCo_2_O_4_/NGr was up to 86%, and its performance was stable. The hybrid battery with high efficiency and high performance was prepared by cooperating with the electrocatalytic reaction of MnCo_2_O_4_/NGr and the ultra-capacitive potential [142]. According to the redox reaction of transition metal (M-O-OH→M-O, M=Ni and Co), NiCo_2_O_4_/NiF@C was prepared by carbon-coated nickel foam as the cathode, which showed ultra-high stability after 5000 cycles. The performances of the ORR and OER were improved by virtue of the rich active center and porous interconnecting structure. The material can serve as a hybrid battery with very long cycle life and large capacity [143]. The emergence of hybrid batteries has circumvented the major constraints confronting the traditional zinc–air batteries. In the future, more efficient hybrid batteries should be designed and applied in real life.

In addition to the above hybrid battery, the photovoltaic battery has also proven to be an effective strategy to improve cell cycle efficiencies. Its motive is based on reducing the overpotential of catalytic reactions with the help of the excessive charge carriers obtained from the optical radiation by the p–n junction. Lv et al. strongly coupled Ni_12_P_5_ nanoparticles (NPs) with nitrogen-doped carbon nanotubes (NCNT) to produce a photoresponsive bifunctional electrocatalyst containing a p–n heterojunction, and this yielded an excellent catalytic activity. Among them, the overpotential of the OER was 360mV@10 mA cm^−2^, and the ORR half-wave potential was 0.9 V. Consequently, the roundtrip efficiency of the ZAB battery increased to 64.2%, while the overpotential dropped to 0.68 V, and the cycle stability and catalytic performance were still better than most other dual functional catalysts, even without light. This concept adds a new catalytic mechanism for the development of OER and ORR dual-functional electrocatalysts [144]. Compared with the experimental conditions of light radiation, the alkaline solution at room temperature is a generally suitable experimental electrolyte, but this solution easily causes drying and induces insoluble carbonate deposition, which limits the performance of zinc–air batteries. Therefore, recent research interests are leaning towards high-temperature molten air systems composed of molten carbonate cocrystal electrolytes. For example, KOH is used as an additive to prepare the molten Li_0.87_Na_0.63_K_0.50_CO_3_ electrolyte. Compared with NaOH, KOH does not cause battery polarization. The 150 charge and discharge cycles at 550 °C show a stable state, with a Coulomb efficiency of 94% and an average discharge voltage of 1.08 V, coupled with a high rate performance at a temperature of 7.3 °C. Although this method requires high temperature and consumes a small amount of weight compared with the traditional aqueous electrolyte, it can effectively solve common problems, such as electrolyte evaporation, zinc dendrite production and short service lives. The results show that the corrosion of the air electrodes is prevented by keeping the molten salt electrolytes at lower temperatures, thus prolonging the battery life [145].

There is a need to explore highly efficient ORR and OER bifunctional catalysts to create a fully green cycle to reversibly convert H_2_O into O_2_. For example, a superactive bifunctional catalyst (Mn_x_O_y_/NC) for a nitrogen-doped macro-ring complex combined under pyrolysis and calcination significantly reduced the overpotential at 0.1 M KOH, which is one of the optimal nonmetal-based reversible oxygen electrodes. In the charging process, the instability of the cathode materials led to the degradation of the electrochemical properties. Although the metal oxide maintained the solid-phase structure, there was a decline in the electrical conductivity of the material. Therefore, carbon materials can be considered as dispersed additives and conductive materials. Wang et al. prepared a NiFe LDH/SNC bifunctional catalyst based on the recombination of sulfur and nitrogen co-doped porous carbon with bimetal (Ni and Fe) hydroxide (LDH), where the ORR half-wave potential was 0.736 V, and the overpotential for OER at 10 mAcm^−2^ was as low as 298 mV with ΔE of 794 mV. The open-circuit voltage of the ZAB cathode assembly battery reached 1.36 V, and this excellent performance is attributed to the coupling action between the components [146]. Although NiFe-LDH exhibits an excellent OER performance, the poor stability limits its further application. To improve the performance, Yan et al. prepared a Co@NC-CNTs@NiFe LDH with a three-functional electrocatalyst. The DFT calculation results showed that the unique 3D-layered structure and the synergy between components and nanostructures could effectively enhance the electrocatalytic activity. More importantly, the constructed self-powered water cracking system has practical application potential [147]. Based on the NiFe LDH and 3D multiphase reaction interface, Wan et al. proposed the “air breathing” strategy by using perfluorosulfonic acid (PFSA)-PFC nanoemulsion immersion FeNi LDH, which could increase the reaction interface and promote the transmission process in air cathode. The resulting electrochemical performance is significantly better than the traditional air electrode [148].

Transition metal complexes of the conductive material macroloop ligands were found to serve as key intermediates to stabilize the high-priced metal ions. For example, the Co/(NiCo)Se_2_, synthesized via the cobalt-based MOF (ZIF-67) as a template, achieved dual functions through the synergy between the special structure and the multicomponent selenide. The unique structure accelerated the electrolyte penetration and ion transmission, improving the volume expansion phenomenon during charging and discharging, and shortening the ion diffusion length towards improving the electrochemical performance. After 80 cycles, the specific capacity reached 497 mA hg^−1^, and the current density was 0.2 Ag^−1^, proving that the material had a good cycle stability [149]. To sum up, the zinc–air battery is expected to replace the common neutral battery as a device with extremely high energy storage and as a power supply in the field industrial park. It can also be used to prepare electronic products such as watches and invest in research in emerging fields such as new energy vehicles, as shown in Table 1.

## 7. Summary and Prospect

In this paper, the research progress of non-precious metal (Fe, Co and Ni)-based ORR/OER catalysts for zinc–air batteries were reviewed. Firstly, the reaction mechanisms of OER and ORR were introduced, and then, the methods to optimize the free energy of the oxygen intermediates and reduce the theoretical overpotential were explored. Finally, the reaction mechanisms of the catalysts were thoroughly described through advanced in situ testing technologies. In the design and synthesis of the catalysts, non-noble metal catalysts and carbon material or other types of catalyst composites, synergistic effects between the components and the complementing defects were shown to further improve the activity of the catalysts. Further studies on the influence of structural characteristics, such as the dimensions and morphology of materials on a catalytic performance, and the reasonable design of porous materials with high intrinsic activity produced more active sites. Various in situ techniques were used to effectively monitor the catalyst structure and the dynamic evolution of reactant molecules during reaction processes. Combined with DFT calculations, the catalytic reaction mechanisms were deeply explored. In order to accurately infer the dynamic reconstruction processes, the in situ characterization techniques should be closely combined with theoretical calculations for the possible “synergistic effects” or “inhibitory effects” and component reconstruction between different active sites. In the design of the oxygen electrodes, the real reaction environment should be considered rather than the activity change on the electrodes. Secondly, the degradation mechanism and structure–activity relationship between the catalysts and membrane electrodes should be studied. The use of appropriate catalysts in the membrane electrode can effectively improve the charge–discharge performances and the activities and stability of the reactions. There are yet some unresolved problems to be considered for future developments. For example, theoretical models are constructed based on the real structures of the catalysts, the structure–activity relationship between the intrinsic structure of the catalysts and their electrocatalytic activities were studied, and deeper insights were gained for the natural processes of the OER and ORR reactions. The flexible solid-state zinc–air battery has great development potential in the future due to its wearable, safe and portable advantages, but there is a need to develop new electrolytes to improve the battery performance. The performance and cycle life of hybrid batteries are better than those of other batteries. At the same time, the safety and stability are guaranteed, which can be combined with the advantages of two different battery technologies to achieve the best effect. In the future, it can be used for new energy generation, contribute to energy conversion and can fundamentally alleviate resource problems and environmental pollution problems.

## Figures and Tables

**Table 1:** The performances of the recently reported hybrid batteries.

Catalysts	Current Density (mA·cm^−2^)	Final Charge/Discharge Voltage Gap (V)	Cycling Time (h)	Round-Trip Efficiency (%)	η (mV) at 10 mA cm^−2^	Tafel Slope (mV dec^−1^)	References
NiO/Ni(OH)_2_ spheres	1	≈0.60	<88	—	—	—	[140]
Co_3_O_4−x_	5	≈0.75	440	—	330	58	[141]
MnCo_2_O_4_/NGr	1	≈0.66	75	86%	—	—	[142]
NiCo_2_O_4_/NiF@C	5	1.0	2600	—	—	—	[143]
NS@Co_3−x_Ni_x_O_4_/Co_3_O_4_	20	0.904	90	60.4%	310	—	[150]
MnS-Ni_x_Co_1−x_S_2_	5	0.67	200	69%	687	—	[151]
NiCo_2_S_4_/3DNCC	1	0.75	400	—	338	79	[152]
Co_3_O_4_/carbon cloth	1	0.83	100	70%	—	88.3	[153]
Co_3_O_4_/Ni foam	10	1.25	333	70%	—	—	[154]
O-Co_3_O_4_/MCN	1	=1.0	300	—	347	79.4	[155]
O-Co_3_O_4_@MCN	1	0.84	300	—	330	107.7	[156]

## Data Availability

Not applicable.
